# Visualization of zinc dynamics in intact plants using positron imaging of commercially available ^65^Zn

**DOI:** 10.1186/s13007-017-0188-0

**Published:** 2017-05-18

**Authors:** Nobuo Suzui, Yong-Gen Yin, Satomi Ishii, Hitoshi Sekimoto, Naoki Kawachi

**Affiliations:** 1 Takasaki Advanced Radiation Research Institute, National Institutes for Quantum and Radiological Science and Technology, Takasaki, Japan; 2 Faculty of Agriculture, Utsunomiya University, Utsunomiya, Japan; 3 Takasaki Advanced Radiation Research Institute, National Institutes for Quantum and Radiological Science and Technology, 1233 Watanuki, Takasaki, Gunma 370-1292 Japan

**Keywords:** Plant nutrition, Zinc, Radionuclide, Non-destructive imaging, Positron imaging

## Abstract

**Background:**

Positron imaging can be used to non-destructively visualize the dynamics of a positron-emitting radionuclide in vivo, and is therefore a tool for understanding the mechanisms of nutrient transport in intact plants. The transport of zinc, which is one of the most important nutrient elements for plants, has so far been visualized by positron imaging using ^62^Zn (half-life: 9.2 h), which is manufactured in the limited number of facilities that have a cyclotron. In contrast, the positron-emitting radionuclide ^65^Zn (half-life: 244 days) is commercially available worldwide. In this study, we examined the possibility of conducting positron imaging of zinc in intact plants using ^65^Zn.

**Results:**

By administering ^65^Zn and imaging over a long time, clear serial images of ^65^Zn distributions from the root to the panicle of dwarf rice plants were successfully obtained.

**Conclusions:**

Non-destructive visualization of zinc dynamics in plants was achieved using commercially available ^65^Zn and a positron imaging system, demonstrating that zinc dynamics can be visualized even in facilities without a cyclotron.

## Background

Positron imaging is widely used to non-destructively visualize the dynamics of positron-emitting radionuclides in vivo. The most common use of positron imaging is cancer screening by positron emission tomography (PET), which exploits the tendency of fluorodeoxyglucose labeled with the positron-emitting radionuclide ^18^F to accumulate in cancer cells. In addition, PET has been used in medical research to analyze the kinetics of drugs labeled with ^11^C, ^13^N, and ^15^O. Meanwhile, in recent years, positron imaging has been used to study plants in research facilities around the world [1–8]. A recent review article emphasized the advantages of positron imaging for understanding the function of the xylem and phloem [9].

Because the major and minor essential elements for plants have a number of positron-emitting radionuclides, positron imaging is a potentially useful tool for understanding the mechanisms of nutrient transport in plants. The radionuclides typically used for positron imaging of plants are limited to ^11^C, ^13^N, ^15^O, and ^18^F, which are processed using well-established purification methods developed for medical research [3, 9]. We have previously studied positron imaging of minor essential and toxic elements, such as ^64^Cu (half-life: 12.7 h) [10] and ^107^Cd (half-life: 6.5 h) [11–16], using the positron-emitting tracer imaging system (PETIS), a two-dimensional positron imaging system (special resolution: approximately 2 mm). However, this approach involves difficulties in purifying the positron-emitting metal radionuclides in other research facilities. Furthermore, most of these positron-emitting nuclides have short half-lives and are therefore not commercially available. Thus, the implementation of positron imaging for plant research is limited to research facilities with a cyclotron, where positron-emitting radionuclides can be produced through nuclear reactions using an accelerated ion beam.

Zinc is one of the essential elements for all living organisms, including higher plants. Zinc deficiency in crops is one of the most serious problems in food production worldwide [17]. Therefore, it is important to understand how plants regulate zinc transport. In the efforts to elucidate the mechanism of zinc transport, positron imaging of zinc dynamics has been a powerful tool. The positron-emitting radionuclide ^62^Zn (half-life: 9.2 h) has been used to study the dynamics of zinc in intact plants [18, 19]. ^62^Zn decays by electron capture (91.6%) and positron emission (8.4%) and produces the radionuclide ^62^Cu (half-life: 9.7 min). Although the positron emission rate of ^62^Zn is weak, its daughter ^62^Cu decays with a 97.8% rate by positron emission to stable ^62^Ni; therefore, a positron imaging system can obtain images of ^62^Zn with high efficiency. However, because ^62^Cu may migrate differently from ^62^Zn in a plant body, there has been the argument that the positron imaging using ^62^Zn correctly reflects zinc dynamics in plants. Furthermore, zinc imaging using ^62^Zn can only be conducted in facilities with a cyclotron.

In contrast, ^65^Zn (half-life: 244 days) is commercially available, and is therefore frequently used as a zinc tracer in the field of plant science [20]. ^65^Zn decays with a 98.6% probability by electron capture and 1.4% by positron emission to stable ^65^Cu. Because the positron emission rate of ^65^Zn is too low, ^65^Zn has been considered to be unsuitable for positron imaging; however, there has been no direct verification of this claim. Therefore, in this study, we examined whether ^65^Zn can be used to visualize zinc dynamics in an intact plant using a positron imaging system, and to estimate the kinetics of zinc uptake using positron imaging data.

## Methods

### Plant cultivation

To visualize zinc dynamics in the whole plant body, dwarf rice plants were cultivated. Seeds of the dwarf rice cultivar *Oryza sativa* L. cv Waito-C were germinated and each plant was grown in a vinyl pot (12 cm in diameter, 10 cm height) filled with artificial soil (Bonsol; Sumitomo Chemical Co., Tokyo, Japan) in a greenhouse (15-h daytime) for 52 days. From 53 to 83 days after seeding, the rice plants were cultivated in a climate chamber under short-day conditions (9-h daytime) with light intensity of approximately 150 μmol m^−2^ s^−1^ and subsequently (84–103 days after seeding) grown in the greenhouse (15-h daytime). To induce strong dwarfing, the rice plants were flooded with uniconazole P solution at a concentration of 0.1 ppm from 78 to 103 days after seeding. From 104 days after seeding, the rice plants were transferred to the climate chamber and cultivated hydroponically in modified Kimura B solution, which consisted of 0.70 mM (NH_4_)_2_SO_4_, 0.17 mM Na_2_HPO_4_·12H_2_O, 0.27 mM K_2_SO_4_, 0.47 mM MgSO_4_·7H_2_O, 0.37 mM CaCl_2_·2H_2_O, 11 mg L^−1^ FeC_6_H_5_O_7_·nH_2_O (Fe citrate), 0.16 µM CuSO_4_·5H_2_O, 0.15 µM ZnSO_4_·7H_2_O, 0.10 µM Na_2_MoO_4_·2H_2_O, 15 µM H_3_BO_3_ and 4.6 μM MnSO_4_·5H_2_O. The culture solution was renewed every week, and the pH was adjusted again to 5.5 at 2 or 3 days after solution renewal. Rice plants approximately 4-months old were used in the whole-plant zinc imaging experiment.

Additionally, typical rice plants (*Oryza sativa* L. cv Nipponbare) were cultivated hydroponically for four weeks in the modified Kimura B solution and used to evaluate uptake kinetics.

### Whole-plant imaging experiment

**Fig. 1:**
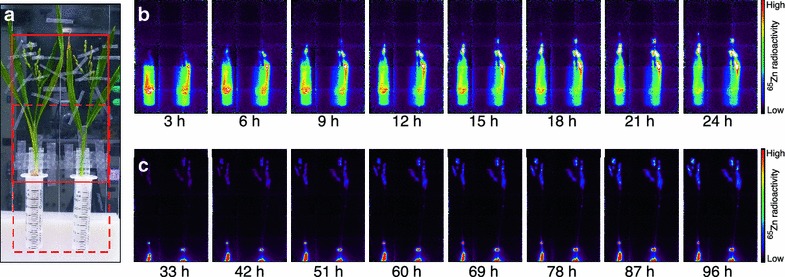
Serial images of ^65^Zn movement in the dwarf rice plants. **a** Photograph of test plants in the experimental apparatus. The *dotted* and *solid rectangles* indicate the field of view of the PETIS during the imaging of the below-ground and the above-ground parts of the plants, respectively. **b** Serial images of the below-ground part (0–24 h). **c** Serial images of the below-ground part (24–96 h).* Each frame* was created from the integration of 18 (**b**) or 54 (**c**) original images collected every 10 min

For whole-plant imaging, the roots of an intact dwarf rice plant were inserted in a 30 mL plastic disposable syringe (Termo Co., Tokyo, Japan), and the shoots were fixed to an acrylic board. The acrylic board, which held two plants at a time, was placed in the field of view of the PETIS (a modified PPIS-4800 positron imaging system; Hamamatsu Photonics, Hamamatsu, Japan) (Fig. 1a). Each syringe was supplied with 30 mL of 0.5 mM CaCl_2_ containing 0.1 µM ZnSO_4_ (3 nmol in total) labeled with 444 kBq of ^65^Zn (22 pmol), which was purchased from RIKEN (Wako, Japan), and 2 mM 2-(N-morpholino)-ethanesulfonic acid. The movement of ^65^Zn in the below-ground parts of the plants, including the roots and the shoot base, was monitored by the PETIS every 10 min for 24 h. Then, the PETIS was moved to the top of the plants and the ^65^Zn movement in the above-ground parts was monitored every 10 min for 72 h. In the first 48 h of the imaging experiment (which lasted 96 h in total), the roots were fed with 0.5 mM CaCl_2_ containing ^65^Zn (the feeding step) and in the last 48 h, they were transferred into Kimura B solution (the chasing step). The solutions were maintained at the same level by supplying 0.5 mM CaCl_2_ or Kimura B solution, which does not contain Zn^+^ with the siphon method, as described by Fujimaki et al. [11]. All imaging experiments were conducted in a growth chamber with continuous light at a density of 400 μmol m^−2^ s^−1^.

The time course data of the zinc amount (mol) in the regions of interest in the images were calculated by the values of the signal intensity (cps) extracted using the NIH Image J 1.50 software (http://rsb.info.nih.gov/ij/), counting efficiency of the system (cps Bq^−1^) and specific radioactivity (Bq mol^−1^).

### Evaluation of uptake kinetics

To experimentally determine the uptake kinetics of zinc, the roots of six non-dwarf rice plants were inserted in a transparent acrylic root box specialized for direct root imaging [12] (Fig. 3a). Each compartment of the root box was supplied with 14 mL of 0.5 mM CaCl_2_ containing different concentrations (0.1, 0.25, 0.5, 1, 2.5 and 5 µM) of ZnSO_4_, labeled with 124 kBq (6 pmol) of ^65^Zn and the ^65^Zn movement from the solution to the roots was monitored by PETIS for 7 h. The solution was continuously stirred with gentle aeration in order to maintain a uniform composition in each compartment of the root box. The uptake velocities for the various zinc concentrations were calculated every hour using the imaging data acquired from the individual rice plants. The obtained substrate velocity data were fitted with the modified Michaelis–Menten equation according to Claassen and Barber [21]:

1 $$I{\text{n}} = \frac{V\hbox{max} \times c}{{K{\text{m}} + c}} - E$$

where, *I*n*, c and E* are the net uptake velocity, substrate concentration and the efflux velocity, respectively. *V*max, *K*m and *E* value were estimated by least-square regression using the python SciPy library (http://www.scipy.org/).

## Results

The tracer solution containing ^65^Zn was administered to dwarf rice plants and the dynamics of ^65^Zn in intact whole plants was monitored by PETIS (Fig. 1a). As a result, obvious clear serial images of ^65^Zn distributions from the root to the panicle were successfully obtained for 96 h (Fig. 1b, c). The counting efficiency of PETIS for ^65^Zn was 6.68 × 10^−4^ cps Bq^−1^, which was sufficient to obtain clear serial images of ^65^Zn distributions from the root to the panicle.

**Fig. 2:**
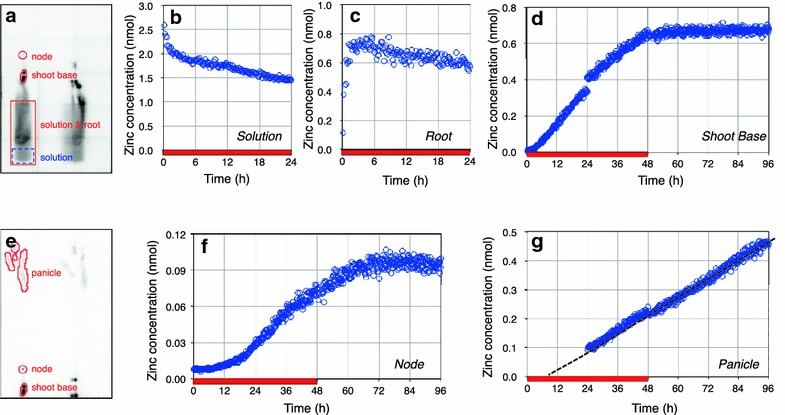
Time course of the amount of zinc in different regions of dwarf rice plants. **a** Examined regions in the underground part of the plants. The *blue dotted rectangle* indicates the region of the solution and the *red solid rectangle* that of the solution and the root. **b** Time course of the amount of zinc in the solution. **c** Time course of the amount of newly acquired zinc in the root. **e** Examined regions of the above-ground part of the plants. **d** Time course of the amount of newly acquired zinc in the shoot base. **f** Time course of the amount of newly acquired zinc in the shoot base. **g** Time course of the amount of newly acquired zinc in the panicle. The *dotted line* indicates a linear approximation of the data. The *red bar* below the time axis represents the duration of the ^65^Zn feeding step, during which 3 nmol of zinc were administered to the plants

Because the amount of non-radioactive zinc labeled with ^65^Zn was calculated by ^65^Zn radioactivity, Fig. 2 represents the time course of “newly acquired” zinc. The amount of zinc in the hydroponic solution decreased rapidly for the first 3 h and more slowly after 6 h (Fig. 2b). In contrast, the amount of newly acquired zinc in the root increased for the first 3 h and then decreased for the remainder of the 24 h (Fig. 2c). Because the shoot base and the node were in the field of view of PETIS during the entire measurement (Fig. 2a, e) time course data could be obtained in these regions for 96 h (Fig. 2d, f). The results show that the amount of newly acquired zinc in the shoot base and the node increased for 48 and 72 h, respectively, and then became plateau (Fig. 2d, f). Although ^65^Zn movement in the panicle was not monitored for the first 24 h, the x-intercept and slope of the linear approximation line fitted to the time course data indicate that zinc was transported from the root and arrived at the panicle 5.3 h after feeding, and monotonically accumulated in the panicle for 96 h at a rate of 5.1 pmol h^−1^ (Fig. 2g).

**Fig. 3:**
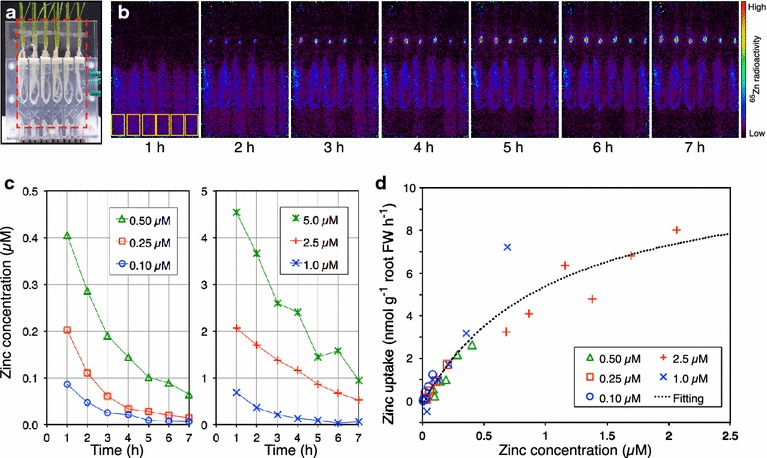
Evaluation of zinc uptake kinetics in non-dwarf rice plants using the ^65^Zn imaging data. **a** Photograph of test plants in the acrylic root box. The *dotted rectangle* indicates the field of view of the PETIS. **b** Serial images of ^65^Zn uptake by rice plants fed with different initial zinc concentrations (*left* to *right*: 0.1, 0.25, 0.5, 1, 2.5, and 5 µM). The *yellow rectangles* indicate the regions examined for the time course analysis. **c** Time course of the zinc concentration in the solution for the different solutions. **d** *Scatter plot* of the uptake velocity as a function of zinc concentration. The *symbols* used for each dataset correspond to the initial zinc concentrations as in **c**. The *dotted curve* represents the modified Michaelis–Menten model fit to the results

We next obtained serial images and quantified zinc uptake in non-dwarf plants for various initial zinc concentrations (0.1–5 µM; Fig. 3b, c). The corresponding zinc uptake velocities were calculated from the decrease in zinc observed in the solution every hour (six values from each plant). The velocity data from the plant fed with 5 µM of initial zinc concentration were excluded from the analysis because the plant was injured during the experimental procedure and the zinc concentration did not monotonically decrease. The obtained substrate velocity data (30 values in total) were fitted by the modified Michaelis–Menten model (Eq. 1) (Fig. 3c) and the values of *V*max, *K*m and *E* value were estimated to be 11.4 ± 1.9 nmol (g root fresh weight)^−1^ h^−1^, 1.1 ± 0.4 µM and 0.15 ± 0.3 nmol (g root fresh weight)^−1^ h^−1^, respectively (R^2^ value: 0.91).

## Discussion

In this study, we demonstrated that ^65^Zn can be used for positron imaging in plants by administering adequate amounts of ^65^Zn and imaging for a sufficiently long time (Fig. 1). In addition, it was demonstrated that the main gamma ray of ^65^Zn (1.1 MeV; 50.6%) does not become noise for positron imaging. These results indicate that non-destructive imaging of zinc dynamics can be conducted even in research facilities without a cyclotron.

In order to capture ^65^Zn movement from the root to the panicle with the restricted size of PETIS field of view, the tested rice plants were artificially dwarfed by treatment with 0.1 ppm of uniconazole Izumi et al. [22] reported that treatment with uniconazole P reduces the fresh weight of both shoots and roots by 39 and 22%, respectively. This result implies that shoot/root ratio is not drastically changed by treatment with uniconazole P. Hence, we sought that the ^65^Zn movement in the dwarf rice plant provides qualitative information on the zinc dynamics in normal rice plant. Our time course measurements revealed that after peaking at approximately 3 h, the amount of newly acquired zinc in the root of the dwarf rice plants decreased (Fig. 2c). On the other hand, in a similar experiment using ^107^Cd, the amount of cadmium in the root of a japonica rice cultivar plateaued within 1 h and increased slightly but did not decrease [12]. Although zinc and cadmium are considered to partially share the same pathway from root to shoot [23, 24], these results suggest that a rice plant usually retains toxic cadmium in the root, whereas it transports zinc to the shoot continuously. The amount of newly acquired zinc in the shoot base became plateau at the end of the feeding step (Fig. 2d), but those in the node and the panicle increased during the chasing step (Fig. 2f, g). These results indicate that zinc absorbed by the root is not accumulated in, but passes through the shoot base to the node and is continuously transported to the panicle in the reproductive stage of rice plants. In addition, we did not observe any ^65^Zn signals in the region of the leaf blade (Fig. 1c). Because zinc is considered to transfer from the xylem to the phloem at nodes and finally accumulate in the panicle [25], these results suggest that the positron imaging of ^65^Zn correctly captures the zinc translocation in rice plants.

Our zinc uptake evaluation results obtained an estimated *K*m value of 1.1 µM, whereas the previously reported value was 5.5 µM [26]. The difference may be caused by the lack of zinc deficient treatment before measurement and by the lack of velocity data at higher substrate concentrations than 2.5 µM in this study. Therefore, our estimation of *K*m using positron imaging data has room for improvement. This approach has the advantage of obtaining several substrate-velocity data measurements from one plant without continuous sampling. Principally, the uptake kinetics of a nutrient element can be estimated from the depletion curve of one plant [21]. The development of this analytical method makes it possible to estimate the *K*m value from an individual plant.

## Conclusions

To the best of our knowledge, this is the first report to visualize zinc movement in living plants using commercially available ^65^Zn and a positron imaging system. Thus far, short-lived ^62^Zn has been used in developing a cancer screening agent for PET [27]. Although ^65^Zn cannot be administered to humans because of its long half-life, it could be applicable to the kinetic analysis of drugs in small experimental animals if close attention is paid to avoid radioactive contamination in laboratories. Therefore, positron imaging of ^65^Zn can be useful not only for the study of zinc dynamics in plants but also for the study of the kinetics of drugs labeled with ^65^Zn in animals.

## Abbreviations

PET: positron emission tomography
PETIS: positron-emitting tracer imaging system
